# Digital Care Horizon: A Framework for Extending Health Care Through Digital Transformation

**DOI:** 10.1016/j.mcpdig.2023.05.005

**Published:** 2023-06-10

**Authors:** Lindsey M. Philpot, Sagar B. Dugani, Abhinav Singla, Meredith DeZutter, Jon O. Ebbert

**Affiliations:** a Department of Medicine, Mayo Clinic, Rochester, MN; b Epidemiology, Department of Quantitative Health Sciences, Mayo Clinic, Rochester, MN; c Kern Center for Science of Health Care Delivery, Mayo Clinic, Rochester, MN

## Abstract

The population needing health care services grows faster than the management capabilities of our current health care delivery models. Patients journeying through our current health care systems receive a spectrum of services, often imperfectly matched to medical needs. We describe a framework of the Digital Care Horizon to accelerate digital transformation from the perspective of a health care delivery system. We describe service delivery models across the horizon, discuss potential challenges and partnerships to facilitate the digital extension of health care, and mention concepts beyond the current horizon.

Digital health encompasses technology-enabled services to advance patient health and well-being. Digital health includes the following: (1) telemedicine and telehealth, enabling patient and health care provider engagement through telephone and video; (2) digital tools, monitoring, aggregating, and sharing patient data with health care providers; (3) mobile health (mHealth), allowing patient access to health care information and expertise through applications for smartphones and tablets; and (4) health information technology, empowering providers to use algorithms and electronic health records (EHRs) to identify, order, and deliver health care services. Digital health has the potential to improve access to health information and expertise, permitting patients health care customization, cost reduction, and the opportunity to have access to omnichannel experiences that meet their medical and social needs.

Within our traditional health care system, patients receive a spectrum of services from incomplete care to overmedicalization, often imperfectly matched to their true needs.^1^ Patients increasingly access digitally enabled health care from their homes and on their mobile devices. New technologies and services create opportunity for medical expertise existing in traditional health care institutions to be disseminated through the new digital frontier and within multiple avenues of care delivery. However, traditional health care organizations may be challenged with ideation, creation, and execution of these digital transformations.

We describe a framework to guide digital transformation through the extension of health care services beyond traditional care settings. We propose the “digital care horizon” (Figure) with centrally sensitized chronic pain as a case example and describe our care models within each domain as embodiments of this framework for change (Table). Radiating from our traditional models of care, the intensity of health care services can be tailored to patient need by leveraging digital tools and services and by offering multiple digital services that can wrap a patient in the care most needed. Digitally transforming how we deliver care will enable more efficient use of health care services, thereby improving both patient experience and total cost of care.

**Figure:**
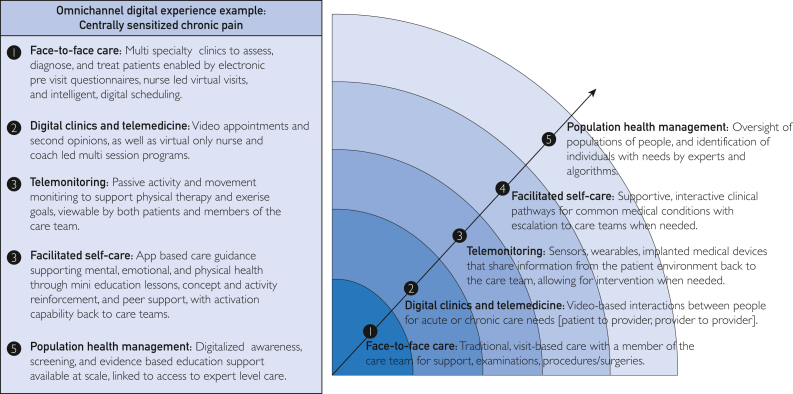
Digital care horizon and example capabilities for patients experiencing chronic pain conditions across the digital care horizon.

**Table:** Current Capabilities and Potential Future Directions in Extending Health Care Services Using Digital Technologies

Care Model	Current Capabilities	Beyond the Horizon
Face-to-face care	• Video and telephone-based previsit consultations • Electronic information gathering from patients before visits • Video and online patient education courses • Augmented/virtual/mixed reality to facilitate patient learning, sedation, and care	• Interactive, digital wayfinding to help patients and caregivers navigate clinical spaces • Intelligent documentation and billing to allow providers more time with patients
Digital clinics and telemedicine	• Video-only visits into patients home or residential setting • Health care facility-to-facility virtual visits with patients and providers at different facilities • Asynchronous electronic consultations (eConsults) between health care providers	• Technology-enabled multispecialty disease boards • Provider-to-provider connection across institutions
Telemonitoring	• Remote monitoring of vital signs • Activity and motion tracking • Medication adherence monitoring • Patient entered information • Decision trees and escalation algorithms	• Device-agnostic systems that use commercially available and medically directed data • Intelligent systems responding to patient need based on clinically and contextually appropriate care guidelines
Facilitated self-care	• Interactive plans for medication management, behavior change, and procedural preparation • Symptom checkers linked to face-to-face and virtual appointments	• Care programs capable of intelligently collating medical needs for patients experiencing multiple conditions into unified care plans that are simple to understand and follow
Population health	• EHR enabled prevention, screening, and ordering • Digital patient education available at scale • Close partnership between public health agencies and health care organizations	• Improved AI algorithms that match patients with services and tools for low acuity needs • Technology-enabled care communities emerge for patient-to-patient, caregiver-to-caregiver, and patient-to-provider support • Internet-of-thing enabling just-in-time information that is consistent and precise, and triaging patient on the basis of need

AI, artificial intelligence; EHR, electronic health record.

### Digital Care Horizon

#### Face-to-Face Care

Traditionally, health care systems have been based on face-to-face models of care whereby clinical team members (physicians, nurses, advanced practice providers, and others) have scheduled visits (in the outpatient setting) or interactions (in the hospital setting) in shared physical spaces.^2^ These models foster connection between people, allow for medical examinations and procedures, and are the most enabled by current payment models. Current face-to-face models can be enhanced through virtual interactions, informed by the remote collection of data outside of the office visit, and augmented through new technologies such as augmented, virtual, and mixed reality to aid in learning and healing, thereby extending the care episode beyond traditional appointments and outside of the current capabilities.

##### Care Model

We have digitally extended our face-to-face models into the spaces before and after patient visits. When patients first approach care at one of our campus locations, they are requested to provide information on previous diagnoses, procedures, and images and on their goals of care. This information is used by allied health and nursing to arrange itineraries for on-site visits and to determine whether a patient may benefit from a virtual previsit.^3^ Information is also requested regarding their social determinants of health. Our clinical teams use this information to understand the environment from which a patient arrives and to arrange for nonmedical services.^4^ Once an episode of care is completed on site, our care teams also digitally extend patient support into the postvisit space through nurse-led individual and group educational programming.^3^ These models extend the “episode of care” beyond the face-to-face visit advancing understanding of patient need, increasing visit efficiency, and supporting the patient through their medical journeys.

#### Digital Clinics and Telemedicine

The coronavirus disease-2019 (COVID-19) pandemic, declared as a public health emergency in March 2020, accelerated the digital transformation of health care.^5^ Many brick-and-mortar practices transitioned care processes to virtual interactions in the face of declining face-to-face appointments and the need to safeguard against COVID-19 transmission.^6^ From 2019 to 2021, the use of telemedicine for office-based physicians increased from 15% to 86%. In 2021, 91% of primary care physicians, 87% of medical specialty physicians, and 75% of surgical specialty physicians used telemedicine.^7^ To expedite the awareness and adoption of digital care, the US Department of Health and Human Services facilitated changes by reimbursing digital interactions at parity with face-to-face interactions. Early in the pandemic, telemedicine was driven by interests to reduce viral transmission; currently, the use of telemedicine is driven by access.^8^ Nationally, workforce shortages in rural settings outpace those in urban settings,^9^, ^10^, ^11^ creating a distinct need for new models of care for medically complex patients in rural settings.

##### Care Model

Within our rural-serving primary care practices, we have virtually empaneled a large population of patients with the highest medical need. We deployed clinical workflows for 2 types of visits to provide telecare: (1) clinic to home and (2) clinic to clinic. With the patient in the rural clinic and the provider at a remote site with video connection through the EHR, we can obtain nurse-acquired vitals along with radiology and laboratory testing to support diagnosis and management. For this type of management to be sustainable and ensure a high level of safety, we have also introduced advanced remote diagnostics tools. From a hospital perspective, digital tools have been used to facilitate postdischarge transition of care through the Postdischarge Early Assessment with Remote video Link (PEARL)^12^ initiative. Through PEARL, launched in Rochester and expanded to rural hospital sites, advanced practice providers conduct a video visit 2-5 days postdischarge and review new and long-term medications, self-management plans, and home supports. Patients reported benefitting from the video visit (agree: 77.3%), expressed interest in future video visits (agree: 70.0%), and would recommend video visits to friends or family (agree: 82.3%). As more patients and practices adapt to the use of telemedicine initiatives, it will be important to provide seamless care outside traditional brick-and-mortar structures.

#### Telemonitoring

Telemonitoring is a broad category including ongoing collection of vital information as a measure of patient status.^13^ Remote monitoring, the expansion of telemonitoring capabilities into patient environments, includes continuous sensors, wearable and handheld devices, and implanted medical devices. The mix of passive and active data collection, linked to care teams able to respond immediately to patient need, expands the reach of medical practices directly into spaces where patients reside. Telemonitoring has been associated with reduced mortality and improved disease self-management.^14^

##### Care Model

Mayo Clinic Center for Digital Health has enabled a technology framework allowing embedded decision trees and advanced logic to drive nursing teams to monitor and respond to remote monitoring sensors outside the walls of health care facilities.^15^ To serve patients with serious and complex medical needs, we developed the Remote Monitoring to Enhance Timely Hospital Dismissal (REMODi) program in partnership with the Department of Nursing and Center for Digital Health.^16^ This program has enrolled episode-based remote monitoring programs for patients with serious and complex conditions, such as cirrhosis and acute kidney injury, and for patients undergoing life-altering operation through pancreatectomy, who are leaving the hospital. Patients are monitored by virtual-first nursing staff who are skilled in remote patient monitoring, with workflows enabling escalation into our specialty nursing and provider teams to instantly connect patients with the expertise needed. Telemonitoring solutions allow care teams to extend health care services into patient residential settings, allowing for healing in environments more comfortable for patients and caregivers.

#### Facilitated Self-Care

Health has been defined as “the ability to adapt and self-manage in the face of social, physical, and emotional challenges.”^17^ Business sector evolution of self-management, such as automated teller machines and airline booking^18^ have heightened patient expectations of health care services.^19^ Facilitated self-care in health care empowers patients and caregivers to manage health-related tasks with the assistance of digital guidance and feedback; several examples are currently available in the iOS and Android application stores.^18^ Current tools enable weight reduction and management, sleep monitoring and feedback, and well-being activities such as guided meditation and resilience training. Barriers to self-care among patients experiencing complex medical conditions include lack of self-care knowledge and lack of confidence in aspects of self-care, difficulty in making and maintaining lifestyle change(s), and lack of social and health care provider support for self-care.^20^^,^^21^ Current technological capabilities are able to help patients build self-efficacy and knowledge regarding their condition, can use aspects of motivational theories to encourage lifestyle change, and can provide peer support and linkage to members of the health care team to facilitate self-care. Provider-directed facilitated self-care products are currently limited but would offer advantages of protocols developed by clinical experts for complex medical conditions and linkage to clinical teams.

##### Care Model

To enable health self-management and to aid procedural preparation, Mayo Clinic has developed interactive medical care plans leveraging medical expertise translated into digitally delivered clinical workflows.^22^^,^^23^ Care plans are enabling self-care of survivors of breast cancer to monitor for suggestion of recurrence, prepare for colonoscopy, and manage chronic migraine through medications and lifestyle. Pilot studies of a program established for chronic insomnia disorder observed favorable patient engagement and program efficacy.^23^ Patient-centered research underpinned the following key design principles driving the creation of our facilitated self-care tools: (1) responsive with customized goals, treatment strategies, and level of interaction; (2) personalized to patient input to support engagement; and (3) knowledgeable about the clinical course and management of medical conditions to drive applicable content accessible “just-in-time.” Facilitating self-care through digital tools will allow clinical teams to extend medical expertise to moments when patients need them the most and within a format that meets patient needs.

#### Population Health

Population health management, or the oversight of populations of people and their health outcomes, such as the identification of individuals in need of social and medical services,^24^ continues to evolve as technology advances and patient expectations change. Extension of care from health care systems to population health models through digital enablement creates opportunities to disseminate evidence-based expertise and to facilitate screening for serious and complex medical conditions. The availability of continuous interconnection through current technologies, such as social media, enables easier communication with patients.^25^ Communication across these media create awareness, affirmation, and support, which has been traditionally hard to achieve in several patients.^26^ With the involvement of institutions continuously generating scientific evidence, electronic educational campaigns can be leveraged as a dissemination strategy. In addition, through incentivization within the American Recovery and Reinvestment Act (2009), many health care organizations have adopted EHRs as a primary source of medical documentation. The digitization of these medical records can be leveraged to screen large amounts of patients to schedule routine preventive tasks, to identify those who may most benefit from clinical intervention, and to “push” diagnostic testing directly to a patient’s home.

##### Care Model

Patients with serious and complex medical conditions often struggle to identify reliable health information. To provide patients with a single place to warehouse reliable health information, our clinical teams have created evidence-based guidance within a web-based platform incorporating video, illustration, and text-based content in addition to interactive games to reinforce key constructs.^16^ These resources are available within our EHR and are made available to >50 new patients a week. Mayo Clinic has also leveraged the EHR to screen patients at risk for poor outcomes related to COVID-19 to receive monoclonal antibody therapy^27^ and for patients with specific diagnoses in need laboratory testing, such as hypertensive patients receiving angiotensin-converting enzyme inhibitors or diuretics who need renal function and electrolyte testing. Health care organizations have an integral role in managing population health, creating accessible information to be used at scale and leveraging tools to identify and connect with patients requiring health care services.

## Discussion

Although several of the care models discussed within the digital care horizon have been piloted or implemented within pockets of care settings or patient populations, broad implementation has been limited. Digitally enabled care models face numerous challenges. First, internet and digital access has been purported to be a “super” social determinants of health, and caution must be taken to ensure development of equitable care models. Digital inclusivity and enablement require health care organizations to work with partners to facilitate internet access, enable use despite digital literacy and readiness and create accessible content across languages and cultures. Second, digital care models must also work for health care providers and not increase “desktop medicine,” worsen burnout, or contribute to attrition. There are likely new roles to serve patients through expertise in patient identification and enrollment within digital solutions, and in digital program monitoring, among others. Payment models, informed by total cost of care from a population health lens and relevant outcomes that are patient-centric will be required to advance current care models into the care models most needed by patients and care teams alike. Finally, it will be imperative to ensure smooth transitions between the levels of the digital care horizon. We will need to leverage experiences and create new insights to determine when a patient can safely be monitored at home or can transition into and out of facilitated self-care models.

Strategic partnerships will be a key component of future success in bridging the health care digital divide. Partnership with patients from all social, racial, and ethnic backgrounds will be essential to inform and cocreate solutions meeting the needs of all. Collaboration with value-aligned technology companies to create solutions while protecting patient privacy will be critical to retain patient trust. Strong relationships between institutional information technology groups and clinical teams will ensure that digital tools fit seamlessly into existing workflows and avoid additional clinical burden. Alliances with public and private payers will be necessary to advance payment models supporting hybrid “bedside-webside” care models that are in alignment with the care goals of patients and people who provide care for them.

Models advancing our ability to extend care to those with serious and complex medical needs through digital platforms are needed. Moreover, omnichannel digital experiences for patients allowing for personalized care based on medical and social need and ability are also needed. We have drafted the digital care horizon as a means to think about opportunities for extension from the perspective of a health care system to help address these needs. There are frameworks from the technology, device, and individual program spaces and those endorsed by the World Health Organization and the Department of Health and Human Services, which provide those who are designing digital health support tools and programs that extend care to bridge the digital divide.

## Potential Competing Interests

Dr Ebbert receives consulting fees from K Health, SOAP Health, and EXACT Sciences paid to Mayo Clinic. Dr Ebbert receives consulting fees from Applied Aerosol Technologies and MedInCell. The funders had no role in study design, data analysis and interpretation; in writing of the manuscript; and in the decision to submit the manuscript for publication. The findings and conclusions do not necessarily represent the views of the funders.
